# Relationships between brain functional connectivity and resting cardiac autonomic profiles in functional neurological disorder: A pilot study^☆^

**DOI:** 10.1016/j.nicl.2026.103996

**Published:** 2026-04-22

**Authors:** Cristina Bleier, Andrew J. Guthrie, Jessica Ranford, Julie MacLean, Ellen Godena, Julie Maggio, Sara A. Finkelstein, Ibai Diez, Christiana Westlin, Karen S. Quigley, David L. Perez

**Affiliations:** a Mass General Brigham Department of Neurology, Massachusetts General Hospital and Brigham and Women’s Hospital, Harvard Medical School, Boston MA, USA; b Athinoula A. Martinos Center for Biomedical Imaging, Massachusetts General Hospital, Harvard Medical School, Boston, MA, USA; c Department of Occupational Therapy, Massachusetts General Hospital, Boston, MA, USA; d Department of Physical Therapy, Massachusetts General Hospital, Boston, MA, USA; e Computational Neuroimaging Lab, Biobizkaia Health Research Institute, Barakaldo, Spain; f Ikerbasque Baske Foundation for Science, Bilbao, Spain; g Center for Inflammation Imaging, Department of Radiology, Mass General Brigham, Harvard Medical School, Boston, MA, USA; h Mass General Brigham Department of Psychiatry, Massachusetts General Hospital and Brigham and Women’s Hospital, Harvard Medical School, Boston, MA, USA; i Department of Psychology, Northeastern University, Boston, MA, USA

**Keywords:** Functional neurological disorder, fMRI, Heart rate, Interbeat interval, Heart rate variability, Central autonomic network

## Abstract

**Background:**

Functional neurological disorder (FND) is associated with alterations in functional brain networks, yet relationships between peripheral autonomic physiology and brain architecture remain poorly characterized. This pilot study examined associations between cardiac autonomic metrics and resting-state functional connectivity (rsFC) in FND.

**Methods:**

Twenty females with FND and 23 age-matched female psychiatric controls (PCs) completed questionnaires, 10-min resting photoplethysmography recordings, and same-day resting-state fMRI. Interbeat interval (IBI) and heart rate variability (HRV) metrics were extracted. Whole-brain rsFC was quantified using weighted-degree [centrality]. Within-group analyses tested associations between cardiac autonomic metrics and weighted-degree rsFC separately in FND and PC cohorts, adjusting for age, head motion, and antidepressant/β-blocker use – while applying a cluster-wise correction.

**Results:**

Cardiac (IBI and HRV) metrics did not differ between FND and PC cohorts, and these metrics did not correlate with FND symptom severity, somatic symptom burden, affective symptoms, or childhood trauma. In FND, shorter IBI (i.e., faster resting heart rate) correlated with higher weighted-degree rsFC in bilateral supplementary motor area (SMA) and right precentral/superior frontal regions, whereas lower HRV primarily correlated with higher weighted-degree rsFC in the bilateral SMA, mid-cingulate cortex, and right amygdala, anterior insula, and lateral orbitofrontal cortex. In PCs, autonomic–rsFC associations were more spatially restricted to the bilateral anterior/mid-cingulate and SMA.

**Conclusion:**

In FND, individual differences in resting autonomic physiology related to the centrality of brain areas that are part of the central autonomic, salience, and allostatic-interoceptive networks. These findings suggest that the relationship between autonomic physiology and network architecture may be important in FND.

## 1 Introduction

Functional Neurological Disorder (FND) is a prevalent, costly, and potentially disabling condition at the intersection of neurology and psychiatry (Aybek and Perez, 2022, Hallett et al., 2022, O’Mahony et al., 2023). Epidemiologic studies demonstrate that FND represents a substantial proportion of outpatient neurology referrals, and is associated with $2 billion annually in emergency department and acute inpatient hospital expenditures in the United States (Finkelstein et al., 2025, Stephen et al., 2025). Clinically, patients with FND exhibit heterogeneous combinations of motor, sensory, and cognitive symptoms that contribute to reductions in health-related quality of life (Anderson et al., 2007, Jones et al., 2016). Concurrent mood, anxiety, and trauma-related symptoms are also common in this population (Butler et al., 2021, Gray et al., 2020). Importantly, clinical practice has shifted from FND as a diagnosis of exclusion to identifying positive “rule-in’’ examination signs (e.g., tremor entrainment, Hoover’s sign), allowing for expedited diagnosis and earlier treatment (LaFrance et al., 2013, Perez et al., 2021a). These advances have catalyzed renewed interest in clarifying the neural circuits and other physiological mechanisms implicated in the pathophysiology of FND.

Resting-state functional MRI (rs-fMRI) studies in FND have identified altered intrinsic brain organization across multiple distributed large-scale brain networks (Baizabal-Carvallo et al., 2019, Homayoun et al., 2025, Perez et al., 2021b, Yeo et al., 2011). Several investigations converge on alterations in the salience, somatomotor, default mode, and frontoparietal networks (Li et al., 2015a, van der Kruijs et al., 2012, Weber et al., 2022, Wegrzyk et al., 2018, Westlin et al., 2025a). In particular, FND cohort studies identify stronger coupling between regions of the salience/ventral attention network—including the anterior insula, dorsal anterior cingulate cortex, and the amygdala—and motor control areas (Demartini et al., 2021, Diez et al., 2019, Li et al., 2015a, Li et al., 2015b, Morris et al., 2017, Seeley, 2019, van der Kruijs et al., 2012). Whole-brain functional connectivity analyses further show increased crosstalk [i.e., *between-network* connectivity] among salience, somatomotor, and default mode networks, the magnitude of which correlated with individual differences in functional somatic symptom severity (Westlin et al., 2025a). Relatedly, altered connectivity patterns in FND implicate multimodal integration hubs—including the anterior insula, anterior and mid-cingulate cortices, and temporoparietal junction—brain areas involved in integrating interoceptive, affective, and cognitive signals with motor control ones (Sepulcre et al., 2012, Shackman et al., 2011). Many of these salience network and multimodal integration hubs—especially the anterior insula, anterior/mid-cingulate cortex, and amygdala—are also components of the central autonomic network (CAN) (Benarroch, 1993, Cechetto and Shoemaker, 2009, Quadt et al., 2022, Saper, 2002) and the allostatic-interoceptive network (Kleckner et al., 2017, Zhang et al., 2025). Within the CAN and allostatic-interoceptive network, distributed cortical, limbic, midbrain, and other brainstem regions coordinate autonomic regulation. This anatomical and functional overlap suggests that contextualizing relationships between resting-state functional connectivity (rsFC) and cardiac autonomic metrics may advance our neurobiological understanding of FND.

In FND, characterization of cardiac autonomic profiles is also an area of active research. Individuals with paroxysmal FND events frequently endorse cardiac autonomic symptoms (e.g., racing heart rate [HR]) as a bodily ‘warning sign’ prior to onset of an episode (Goldstein and Mellers, 2006, Indranada et al., 2019). This phenomena has been termed “panic attack without panic” – characterized by individuals experiencing some of the physical symptoms associated with panic attacks without a panic-related emotional experience (Goldstein and Mellers, 2006, Jungilligens et al., 2022). Although findings across cohorts are heterogeneous (particularly in adults), several observations point towards altered cardiac autonomic profiles in FND relative to controls (Bakvis et al., 2009, Kozlowska et al., 2018, Kozlowska et al., 2017a, Kozlowska et al., 2015, Maurer et al., 2016, Ponnusamy et al., 2011, Radmanesh et al., 2020, Reinsberger et al., 2012, Sawchuk et al., 2020). A 2022 *meta*-analysis by Paredes-Echeverri et al. (2022) found higher resting HR in patients with FND across pediatric and adult cohorts compared to healthy controls (HCs), along with a tendency towards reduced heart rate variability (HRV) at rest, which commonly concurs with faster resting HR (Paredes-Echeverri et al., 2022). Autonomic metrics in some studies have also been shown to co-vary with clinically meaningful variables — including symptom severity, subjective distress, anxiety, and illness duration — further underscoring the importance of autonomic profiles in FND (Allendorfer et al., 2019, Kozlowska et al., 2015). In the pediatric FND literature, several brain imaging studies have probed the neural correlates of cardiac autonomic profiles – primarily measured through resting HR. For example, differences in somatosensory cortex-to-cerebellar network connectivity were related to resting HR values in pediatric FND (Rai et al., 2022). RsFC profiles across insular sub-regions also exhibited trend-level correlations with resting HR (Walpola et al., 2025). To our knowledge, no studies in adults with FND have investigated relationships between resting cardiac autonomic metrics and resting-state functional brain organization.

In this pilot study, we examined out-of-scanner cardiac autonomic metrics at rest in females with FND compared to age-matched female psychiatric controls (PCs). We focused on cardiac autonomic measurements of IBI (inverse of HR) and HRV, which are metrics related to resting cardiac arousal. We subsequently investigated how individual differences in IBI and HRV related to whole-brain, weighted-degree [global centrality] derived from rs-fMRI – with analyses performed separately in FND and PC cohorts. Weighted-degree centrality reflects the global influence of a region within the brain’s overall functional architecture by capturing the extent of its functional connectivity to all other regions (Diez et al., 2021). This whole-brain summary measure allows us to test whether individual differences in resting cardiac autonomic metrics relate to large-scale patterns of rsFC across the brain, rather than focusing on isolated pairwise connections or *a priori* seeds. Given that the CAN and allostatic-interoceptive networks include brain areas implicated in the pathophysiology of FND, we hypothesized that shorter resting IBI (i.e., higher resting HR) and lower resting HRV, two metrics that may reflect greater cardiac arousal, would relate to cingulo-insular and amygdala rsFC profiles. We also hypothesized that a subset of these functional connectivity-autonomic relationships would be distinct in patients with FND compared to PCs, in keeping with partially overlapping and distinct neurobiological mechanisms in these two populations (Diez et al., 2021, Westlin et al., 2025a). Given that medications can influence autonomic physiology, a secondary aim was to explore the impact of antidepressants, beta-blockers, α-adrenergic blockers, and stimulant medications on within-group associations between rsFC and cardiac autonomic profiles.

## 2 Methods

### 2.1 Participants

Twenty female participants with FND [mean age (SD) = 35.9 ± 13.2 years; Table 1] were prospectively recruited from the Massachusetts General Hospital FND Unit between October 2022 and March 2025. Rule-in diagnoses were established based on positive examination signs, semiological features (e.g., tight eye closure at event onset, asynchronous limb shaking movements), and electroencephalogram data, when applicable (i.e., for functional seizures only) (LaFrance et al., 2013). The FND cohort was ‘mixed’, meaning individuals had a range of phenotypes; a transdiagnostic approach was taken given that many individuals with FND exhibit mixed symptoms cross-sectionally and/or develop distinct FND symptoms longitudinally (McKenzie et al., 2011, Tinazzi et al., 2020). The FND cohort included 15 individuals with functional motor disorder and seven people with functional seizures; two participants met diagnostic criteria for both phenotypes (see Supplementary Table 1 for a complete description of participant phenotypes). Exclusion criteria included major neurological comorbidities (e.g., epilepsy, Parkinson’s disease), known brain MRI abnormalities, poorly controlled medical problems with known central nervous system consequences, active illicit substance use disorder, history of psychosis, and/or active suicidality. One additional female with FND was enrolled but excluded following outlier inspection of mean weighted-degree values (defined as having a mean weighted-degree at the whole-brain level exceeding more than 1.5 times the upper or lower interquartile range values). Males with FND were also excluded from this pilot, given the limited number of such individuals who have participated in dual brain MRI and autonomic data collection to date (n = 3). This autonomic-focused pilot study is part of a larger project from which cross-sectional structural MRI and rs-fMRI data have been previously published (Westlin et al., 2025a, Westlin et al., 2025b, Westlin et al., 2025c); no autonomic data for this cohort has been previously published.

**Table 1:** Clinical characteristics of females with functional neurological disorder (FND) versus psychiatric control (PC) cohort.

	Functional Neurological Disorder (n = 20) mean ± SD or N (%)	Psychiatric Controls (n = 23) mean ± SD or N (%)	p-value ^a^
Age (years)	35.9 ± 13.2	33.2 ± 12.4	0.68 (0.68)
FND-motor	15 (75.0%)	−	−
FND-seizure	7 (35.0%)	−	−
SOMS:CD	8.1 ± 6.3	0.17 ± 0.4	<0.001 (0.001)
SDQ-20	32.5 ± 7.6	23.1 ± 5.3	<0.001 (0.001)
PHQ-15	13.6 ± 5.9	8.13 ± 4.4	<0.001 (0.001)
BDI-II	17.0 ± 12.1	15.4 ± 13.3	0.55 (0.66)
STAI-Total	89.4 ± 23.7	80.6 ± 24.4	0.33 (0.44)
PCL-5	23.4 ± 17.9	19.5 ± 12.8	0.28 (0.44)
CTQ-Total	59.8 ± 20.6	53.3 ± 15.8	0.26 (0.44)
SSRI/SNRI use	11 (55.0%)	11 (47.8%)	0.65 (0.68)
Beta-blocker use	5 (25.0%)	2 (8.7%)	0.15 (0.36)
Alpha-adrenergic blocker use	6 (30.0%)	2 (8.7%)	0.08 (0.24)
Stimulant use	5 (25.0%)	9 (39.1%)	0.32 (0.44)

^a^*p*-values correspond to uncorrected statistics, with false discovery rate (FDR)-corrected *p*-values shown in parentheses. For PHQ-15, SDQ-20, BDI-11, STAI, PCL-5 and CTQ, there is missing data on 3 FND participants and 1 PC participant. FND-motor, functional neurological disorder motor subtype; FND-seizure, functional neurological disorder seizure subtype; SOMS:CD, Screening for Somatoform Symptoms-7 Subscale for Conversion Disorder; SDQ-20, Somatoform Dissociation Questionnaire-20; PHQ-15 Patient Health Questionnaire-15; STAI, Spielberg State-Trait Anxiety Inventory; PCL-5, PTSD Checklist 5; CTQ, Childhood Trauma Questionnaire; SSRI/SNRI, selective serotonin reuptake inhibitor/serotonin-norepinephrine reuptake inhibitor. PHQ-15 and STAI scores were normally distributed; between-group differences were evaluated using independent sample *t*-tests. SOMS:CD, SDQ-20, BDI-II, PCL-5 and CTQ scores were not normally distributed and therefore compared using Mann-Whitney U tests. For categorical variables, chi-square tests were applied to SSRI/SNRI use and stimulant use, whereas Fisher’s exact tests were used for beta-blocker use and alpha-adrenergic medication use.

Twenty-three female PCs [mean age (SD) = 33.2 ± 12.4 years] were prospectively recruited through community advertisements. PCs had a lifetime history of depression (n = 19), anxiety (n = 17), and/or post-traumatic stress disorder (PTSD) (n = 8) (Supplementary Table 1). Psychiatric diagnoses were made via the Structured Clinical Interview for DSM-5 (SCID-5). Exclusion criteria for those in the PC group were the same as for those with FND, except they had no diagnosis of FND. Five additional PC female subjects were enrolled but excluded following fMRI weighted-degree outlier inspection (n = 1) and quality inspection of autonomic data (n = 4, see below). All subjects provided informed consent, and the Mass General Brigham Institutional Review Board approved this study.

## 3 Procedure overview

Participants had an in-person study visit at the Athinoula A. Martinos Center for Biomedical Imaging (Boston, MA). Upon arrival, individuals first completed a limited set of psychometric questionnaires (see *Psychometric Characterization*). Autonomic physiology at rest was then recorded using the Empatica E4 wristband under standardized conditions (see *Autonomic Data Acquisition and Preprocessing*). Following autonomic data collection, participants underwent brain MRI scanning the same day (see *MRI Acquisitions and Preprocessing*). Afterwards, participants were emailed a secure electronic link to complete the remainder of study questionnaires.

## 4 Psychometric characterization

On the day of autonomic and rs-fMRI measurements, individuals initially completed two questionnaires: i) the Screening for Somatoform Symptoms-7 Subscale for Conversion Disorder (SOMS:CD), which is a self-report scale of FND symptoms (e.g., paralysis, impaired balance, seizures) experienced over the past week, rated on a 5-point Likert scale (Rief and Hiller, 2003); and ii) the 20-item state portion of the Spielberger State-Trait Anxiety Inventory (Spielberger, 2010). The following additional questionnaires were completed via a secure electronic link after on-site activities: 1) the Somatoform Dissociation Questionnaire-20 (SDQ-20), a 20-item scale evaluating the extent to which core FND symptoms (e.g., paralysis) were experienced over the past year on a 5-point Likert scale (Nijenhuis et al., 1996); 2) the Patient Health Questionnaire-15 (PHQ-15), a 15-item scale measuring how bothersome physical symptoms (e.g., pain, fatigue) were over the past 4 weeks on a 3-point Likert scale (Kroenke et al., 2002); 3) the 20-item trait portion of the Spielberger State-Trait Anxiety Inventory; 4) the Beck Depression Inventory (BDI-II); 5) the PTSD Checklist for DSM-5 (PCL-5); and 6) the Childhood Trauma Questionnaire (CTQ). Four participants had incomplete psychometric data (3 FND and 1 PC).

## 5 Cardiac autonomic data acquisition and preprocessing

***Physiological Measurement.*** Resting autonomic data were collected using the Empatica E4 wristband, a research-grade medical device which collects raw physiological data (CE-certified, No. 1876/MDD; https://www.empatica.com/research/e4/). The device measures blood volume pulse (BVP) from the photoplethysmographic (PPG) sensor, which collects data at 64 Hz through a red-green photodiode system attached to the participant’s left wrist.

Participants were instructed to turn off their mobile devices, sit upright in a comfortable position with their back supported by the chair and hands resting on their lap, and minimize unnecessary movement throughout the recording to ensure good signal quality. The Empatica E4 device was fitted on the participant’s left wrist, and data were streamed in real time to an iOS device running the E4 Connect app. After device placement, researchers visually confirmed adequate BVP signal quality and adjusted strap tension if needed to optimize sensor contact. A button press on the E4 was used to insert a timestamp marking the start of the 10-minute resting-state recording, after which research staff exited the room. The participant sat quietly and alone in a well-lit room during data collection. At the end of the 10-minute period, staff re-entered the room and inserted a second timestamp marking the end of the recording.

***Signal Preprocessing.*** The raw BVP signal obtained from PPG sensors was processed using Kubios HRV Scientific software (Kubios Oy, Finland; version 4.1.2.1; Tarvainen et al., 2014). Beat-to-beat intervals were detected using the software’s built-in pulse detection algorithm (Lipponen and Tarvainen, 2019). All recordings underwent visual inspection to verify beat detection accuracy and identify segments contaminated by noise or motion artifacts; no manual correction of beat detection was performed because only artifact-free segments of data were used (see below).

To allow for physiological stabilization after placement of the E4 device, the first 60 s of recording were excluded for all participants. The remaining data were divided into 40–60 s segments to optimize signal quality and minimize artifact-related loss. We used multiple windows of 40–60 s duration to balance the need for sufficiently reliable estimates per window, where 30–50 s has been reported to be sufficient to provide reliable estimates of HRV at rest (Salahuddin et al., 2007), while also retaining as many uninterrupted time series windows as possible to maximize the amount of data that could be used per participant. Segments containing artifacts flagged by the Kubios software using default parameters were omitted, and a 2-beat buffer was applied before and after all detected artifacts to ensure clean IBI extraction. Following checks for artifactual segments, IBI, HR, and HRV metrics were computed for each valid segment using Kubios and subsequently exported for statistical analysis in SPSS (Version 28). Within Kubios, IBI was defined as the time (in milliseconds) between successive pulse peaks; three HRV metrics were also derived: (1) root mean square of successive differences (RMSSD) or the square root of the mean of squared differences between adjacent IBIs; (2) standard deviation of normal-to-normal intervals (SDNN) reflecting the standard deviation of all IBIs within a segment; and (3) high-frequency (HF) HRV power (0.15–0.40 Hz) via autoregressive spectral analysis of the IBI series.

For each participant, autonomic metrics were averaged across all available high-quality segments obtained within the 10-minute resting recording. Quality control procedures included visual review of raw PPG waveforms and evaluation of the physiological plausibility of the derived respiratory rate (i.e., within the high-frequency HRV band range of 0.15–0.40 Hz). Data quality characteristics are summarized in Supplementary Table 2.

## 6 MRI acquisition and preprocessing

High-resolution anatomical and rs-fMRI data were acquired on the same 3 T scanner using a 12-channel phased-array head coil. A high-resolution T1-weighted magnetization-prepared rapid gradient echo (MP-RAGE) scan was acquired for each subject with the following parameters: 1 mm isotropic voxels; 160 sagittal slices; acquisition matrix size = 256x256; repetition time = 2300 ms; echo time = 2.98 ms; field of view = 256 mm. Resting-state blood oxygen level-dependent (BOLD) scans were acquired using T2*-weighted echo-planar imaging sequences with the following parameters: TR = 3000 ms; TE = 30 ms; flip angle 85; 216 mm FOV; 3 mm isotropic voxels; sequence length = 6 min, 12 s. All participants had two resting-state BOLD scan acquisitions, except for two PC participants, who had one acquisition. During scanning, participants were instructed to remain as still as possible with their eyes open. Bi-temporal foam pads restricted head motion, and earplugs were used to attenuate scanner noise.

Preprocessing was performed using the FMRIB Software Library (FSL v5.0.7; Oxford, UK) and MATLAB R2023a (MathWorks, Natick, MA), following pipelines previously implemented in this cohort (Westlin et al., 2025a). Anatomical (T1-weighted) preprocessing included reorientation to right-posterior-inferior (RPI) orientation, alignment to the anterior/posterior commissure plane, skull stripping, tissue segmentation into grey matter, white matter, and cerebrospinal fluid (CSF), and nonlinear registration to the 3-mm isotropic MNI152 template. Anatomical labeling and regional localization were performed using the Harvard-Oxford cortical and subcortical probabilistic atlases (Desikan et al., 2006).

Rs-fMRI preprocessing included removal of the first four volumes to allow for signal equilibration, slice-timing correction, reorientation to RPI, and within-run realignment using a six-parameter rigid-body transformation. Mean functional images were registered to individual skull-stripped T1-weighted images, followed by nonlinear transformation into MNI space. Additional preprocessing steps included spatial smoothing with a 6-mm full-width at half-maximum (FWHM) Gaussian kernel, intensity normalization, and band-pass temporal filtering (0.01–0.08 Hz). Confound regression removed 12 motion-related parameters (six affine motion estimates and their temporal derivatives), linear and quadratic trends, and five anatomical noise components each from lateral ventricular CSF and white matter masks. Framewise displacement was computed from realignment estimates, and volumes with frame displacement > 0.5 mm were scrubbed [removed] from the data. To ensure comparable data quantity across participants, 120 volumes were retained per subject. For participants with multiple resting-state acquisitions, runs were concatenated, and the first 120 usable volumes were included in the connectivity analyses**.**

## 7 Resting state functional connectivity analyses

Using previously published methods (Diez et al., 2021, Westlin et al., 2025a), we investigated network centrality via whole-brain weighted-degree rsFC, which assesses each voxel’s contribution to the overall resting architecture of the brain. A single grey-matter mask in 3 mm isotropic MNI space was used for all analyses. We generated this using the Human Connectome Project mean grey matter partial volume estimate map with a threshold of 0.2. This mask was applied to all participants, such that weighted-degree was computed over the same set of grey-matter voxels in every subject. We first computed whole-brain rsFC matrices by extracting rs-fMRI time series from all grey matter voxels and computing Pearson correlation coefficients between each voxel pair. Correlation coefficients were Fisher r-to-z transformed prior to weighted-degree computation. Negative correlations were removed due to their controversial interpretation (Qian et al., 2018). Weighted-degree was then computed as follows:

(1) $${\mathrm{WD}}_{i}=\sum_{j=1}^{n}adj\left(i,j\right)$$

where the weighted-degree for voxel *i* (*WD_i_)* represents the sum of that voxel’s connections to all other voxels *j*. Weighted-degree was computed as the sum of all positive correlations, without additional thresholding. This approach avoids arbitrary threshold selection, and has been commonly used in weighted-degree analyses, including prior published work from our group (Diez et al., 2021, Westlin et al., 2025a). The weighted-degree of each voxel was summarized in an adjacency matrix for each participant, which was then transformed into a brain map depicting each voxel’s global rsFC.

## 8 Statistical analyses

### 8.1 Cardiac autonomic physiology and psychometrics

Autonomic physiological metrics were compared between FND and PC cohorts using independent-samples t-tests for normally distributed metrics and Mann-Whitney U tests for non-normally distributed metrics (with False Discovery Rate (FDR) correction applied across autonomic metrics). Associations between cardiac autonomic metrics and psychometric scores were quantified using Spearman rank correlation coefficients, again applying FDR correction. Outliers were identified using the 1.5 x interquartile range (IQR) criterion and removed on a per-metric and per-group basis prior to statistical analyses.

### 8.2 Within-group weighted-degree rsFC

Primary analyses examined the relationship between weighted-degree rsFC and cardiac autonomic metrics separately within the FND and PC cohorts. For each autonomic metric, general linear models (GLMs) tested associations between weighted-degree rsFC and autonomic metrics (i.e., IBI, RMSSD, SDNN, HF-HRV) within each group. Mean HR, while reported for descriptive purposes, was not used as the physiologic metric of interest. Instead, IBI was used (inverse of HR), which has better metric qualities than HR because IBI is more linearly related to the underlying parasympathetic and sympathetic nerve activities that alter the timing of the heartbeat (Berntson et al., 1995, Quigley and Berntson, 1996). Each model controlled for age, mean framewise displacement, SSRI/SNRI use (yes/no), and beta blocker medication use (yes/no) as covariates. Secondary (*post-hoc*) models additionally controlled for α-adrenergic blocker use (yes/no) and stimulant use (yes/no). Resulting t-statistic maps were converted to Z-statistics and corrected for multiple comparisons using cluster-wise correction implemented in AFNI (Cox, 1996). Spatial smoothness was estimated from model residuals within the grey matter mask using 3dFWHMx (with the −acf option) and averaged across residuals. Monte Carlo simulations were performed using 3dClustSim with 10,000 iterations within the same mask. A two-sided voxel-wise threshold (|Z| > 1.96) was applied, with cluster-wise correction at p < 0.05.

### 8.3 Exploratory post-hoc between-group analyses.

To evaluate whether the correlations between weighted-degree rsFC values and indices of resting autonomic tone in participants with FND reflected regional differences as compared to values observed in PCs, we conducted a set of exploratory *post-hoc* between-group comparisons. Specifically, for each cardiac autonomic metric that showed a significant association with weighted-degree rsFC in the FND cohort, we then compared weighted-degree values between FND and PCs within the voxels that survived all primary and *post-hoc* corrections in the main analysis. GLMs were used to compare FND vs. PCs, correcting for age and mean framewise displacement. This exploratory analysis assessed whether weighted-degree rsFC values within autonomic-related regions were higher, lower, or generally the same in FND participants compared with PCs, providing additional context for interpretation of the primary results.

## 9 Results

### 9.1 Demographic and psychometric comparisons

There were no differences between patients with FND and PCs on age, medication use (SSRI/SNRI, β-blocker, α-adrenergic blocker, or stimulant use), or scores on the BDI-II, STAI-Total, PCL-5, and CTQ-Total. However, compared with PCs, the FND cohort had increased SOMS:CD, SDQ-20, and PHQ-15 scores (Table 1).

## 10 Resting cardiac autonomic physiology

Mean HR, IBI, RMSSD, SDNN, and HF HRV values did not statistically differ in patients with FND compared to PCs (Table 2). Additionally, the cohorts were similar in terms of PPG data quality and signal characteristics from Empatica E4 recordings, including the number of segments analyzed per subject, mean segment duration, and total analyzed data duration (Supplementary Table 2).

**Table 2:** Comparison of resting heart rate (HR) and heart rate variability (HRV) metrics between participants with functional neurological disorder and psychiatric controls.

	Functional Neurological Disorder (n = 20) mean ± SD .	Psychiatric Controls (n = 23) mean ± SD .	p-value ^a^
HR (bpm)	78.2 ± 10.9	77.9 ± 15.0	0.68 (0.85)
IBI (ms)	782.3 ± 107.7	777.9 ± 129.1	0.91 (0.91)
RMSSD (ms)	36.8 ± 23.8	27.6 ± 10.5	0.51 (0.85)
SDNN (ms)	39.3 ± 20.9	30.5 ± 12.4	0.24 (0.85)
HF HRV (log)	5.9 ± 1.5	5.6 ± 1.1	0.38 (0.85)

^a^*p*-values correspond to uncorrected statistics, with false discovery rate (FDR)-corrected *p*-values shown in parenthesis. IBI, interbeat-interval; SDNN, standard deviation of normal-to-normal interval; RMSSD, root mean square of successive differences; HF HRV, high-frequency heart rate variability spectral power; bpm, beats per minute; ms, milliseconds; log, natural logarithm transformed values of absolute power of HF band (ms).

In both the FND and PC cohorts, cardiac autonomic metrics showed no significant correlations with psychometric variables (**Supplementary Fig. 1**).

## 11 Associations between cardiac autonomic measures and weighted-degree rsFC

**Fig. 1:**
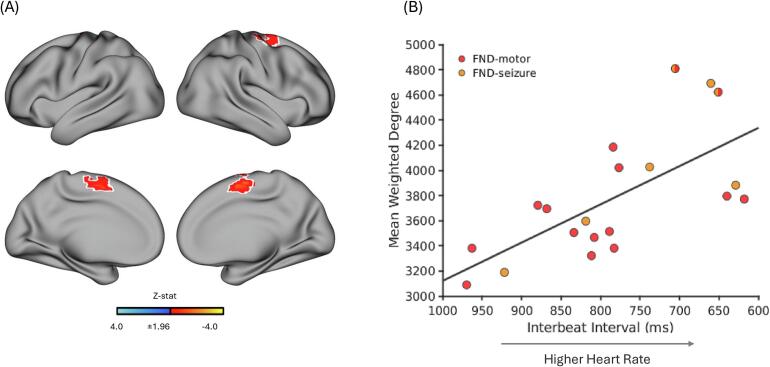
**Weighted-degree resting-state functional connectivity (rsFC) correlated with interbeat interval (IBI) within female patients with functional neurological disorder (FND; n = 20)**. **A**) Adjusting for age, head motion, SSRI/SNRI use (yes/no) and β-blocker use (yes/no), relatively shorter IBI (higher heart rate) was associated with higher weighted-degree rsFC in the bilateral supplementary motor area, right precentral and superior frontal gyri. The white outline marks the subset of voxels that remain significant across *post-hoc* corrections additionally adjusting for α-adrenergic blocker use (yes/no) and stimulant medication use (yes/no). Maps show clusters defined by a voxel-wise threshold of z-statistic > 1.96, corrected for multiple comparisons at the cluster level (*p* < 0.05). The volumetric brain map is shown on the cortical surface for display purposes only. **B**) Scatterplot displays mean weighted-degree values extracted from voxels within the white outlined regions versus IBI (ms). The x-axis is reversed to indicate that shorter IBI corresponds to higher heart rate (arrow). Z-stat, Z-statistic.

Within the FND cohort, shorter IBIs (i.e., higher HR, or greater cardiac arousal) were associated with higher weighted-degree rsFC in the bilateral supplementary motor area (SMA) and the right precentral / superior frontal gyri after adjusting for age, head motion, SSRI/SNRI (yes/no), and β-blocker medication use (yes/no) (Fig. 1). These effects largely remained significant following additional *post-hoc* adjustments for α-adrenergic blocker and stimulant medication use.

**Fig. 2:**
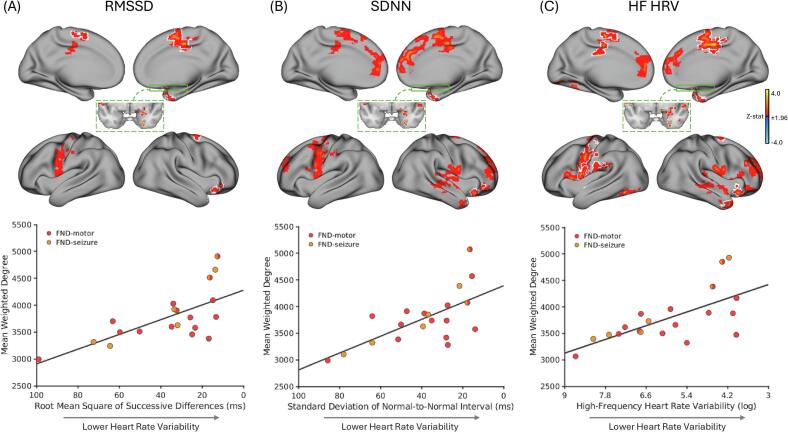
**Weighted-degree resting-state functional connectivity (rsFC) correlated with time- and frequency-domain heart rate variability metrics within female patients with functional neurological disorder (FND; n = 20)**. **A)** Adjusting for age, head motion, SSRI/SNRI use and β-blocker use, lower root mean square of successive differences (RMSSD) values [a proxy of greater cardiac arousal] were associated with higher weighted-degree rsFC in the bilateral supplementary motor area, mid-cingulate cortices, left precentral and postcentral gyri, central opercular cortex, and right orbitofrontal cortex, temporal pole, amygdala, nucleus accumbens, putamen (*top* panel). White outlines indicate regions that remained significant after *post-hoc* analyses additionally correcting for α-adrenergic blocker use (yes/no) and stimulant medication use (yes/no). In the *bottom* panel, the scatterplot displays mean weighted-degree values extracted from voxels within the white outlined regions versus RMSSD (ms). **B)** Adjusting for age, head motion, SSRI/SNRI use and β-blocker use, lower standard deviation of normal-to-normal intervals (SDNN) [a proxy of greater cardiac arousal] was associated with higher weighted-degree rsFC in the bilateral supplementary motor area, anterior and mid-cingulate cortices, paracingulate gyri, medial prefrontal cortices, frontal poles, central opercular cortices, left precentral and superior frontal gyri, and right orbitofrontal cortex, insula and insular-opercular cortex, superior/middle temporal gyrus, temporal pole, and amygdala (*top* panel). White outlines indicate regions that remained significant after *post-hoc* corrections additionally adjusting for α-adrenergic blocker use (yes/no) and stimulant medication use (yes/no). In the *bottom* panel, the scatterplot displays mean weighted-degree values extracted from voxels within the white outlined regions this versus SDNN (ms). **C)** Adjusting for age, head motion, SSRI/SNRI use and β-blocker use, lower values of high frequency heart rate variability (HF HRV) [a proxy of greater cardiac arousal] were associated with higher weighted-degree rsFC in the bilateral supplementary motor area, anterior and mid-cingulate cortices, paracingulate gyri, medial prefrontal cortices, central opercular cortices, left precentral, postcentral, and lingual gyri, and right orbitofrontal cortex, inferior frontal gyrus, frontal pole, insula, superior/middle temporal gyrus, temporal pole, amygdala, thalamus, and putamen (*top* panel). White outlines indicate regions that remained significant after additional post-hoc corrections for α-adrenergic blocker use and stimulant medication use. In the *bottom* panel, the scatterplot displays mean weighted-degree values extracted from voxels within the white outlined regions versus HF HRV (ln-ms^2^). Maps show clusters defined by a voxel-wise threshold of z-statistic > 1.96; corrected for multiple comparisons at the cluster level (*p* < 0.05). Volumetric brain maps are visualized on the cortical surface for display purposes only. Note: in all scatterplots the x-axis is reversed to indicate that lower RMSSD, SDNN and HF HRV values correspond to lower HRV as indicated by the arrow [proxy for greater cardiac arousal]. Z-stat, Z-statistic; RMSSD, root mean square of successive differences; SDNN, standard deviation of normal-to-normal intervals; HF HRV, high frequency heart rate variability.

Within FND participants, lower time- and frequency-domain HRV metric values, potentially a reflection of greater cardiac arousal, were consistently associated with higher weighted-degree rsFC. Specifically, lower RMSSD (lower HRV) correlated with higher weighted-degree in the bilateral SMA, mid-cingulate cortices, left precentral and postcentral gyri, central opercular cortex, and right orbitofrontal cortex, temporal pole, amygdala, nucleus accumbens, and putamen (Fig. 2A). After adjusting for α-adrenergic blocker and stimulant medication use, associations remained significant within the bilateral SMA and right mid-cingulate cortex, orbitofrontal cortex, temporal pole, and amygdala regions.

Similarly, lower SDNN (also reflective of lower HRV) was associated with higher weighted-degree rsFC in the bilateral SMA, anterior and mid-cingulate cortices, paracingulate gyri, medial prefrontal cortices, frontal poles, central opercular cortices, left precentral, and superior frontal gyri, and right orbitofrontal cortex, insula and insular-opercular cortex, superior/middle temporal gyrus, temporal pole and the amygdala (Fig. 2B). After adjusting for α-adrenergic blocker and stimulant medication use, associations remained significant within the right orbitofrontal cortex, temporal pole and amygdala.

Finally, lower HF HRV, associated with greater cardiac arousal, was associated with higher weighted-degree rsFC across the bilateral SMA, anterior and mid-cingulate cortices, paracingulate gyri, medial prefrontal cortices, central opercular cortices, left precentral, postcentral, and lingual gyri, and right orbitofrontal cortex, inferior frontal gyrus, frontal pole, insula, superior/middle temporal gyrus, and temporal pole regions, as well as the right amygdala, thalamus, and putamen (Fig. 2C). After adjusting for α-adrenergic blocker and stimulant medication use, associations remained significant within the bilateral SMA, the right amygdala, temporal pole, insula, orbitofrontal cortex, and the left precentral, postcentral, and lingual gyri.

Notably, within FND participants lower HRV across all three metrics showed significant associations with higher weighted-degree rsFC in the right amygdala, temporal pole, and orbitofrontal cortex that held across all *post-hoc* adjustments. In addition, both lower RMSSD and HF HRV values showed consistent associations with higher weighted-degree in the bilateral SMA across all *post-hoc* adjustments. See **Supplementary Fig. 2** and Supplementary Table 3 for all significant rsFC-autonomic relationships in females with FND.

**Fig. 3:**
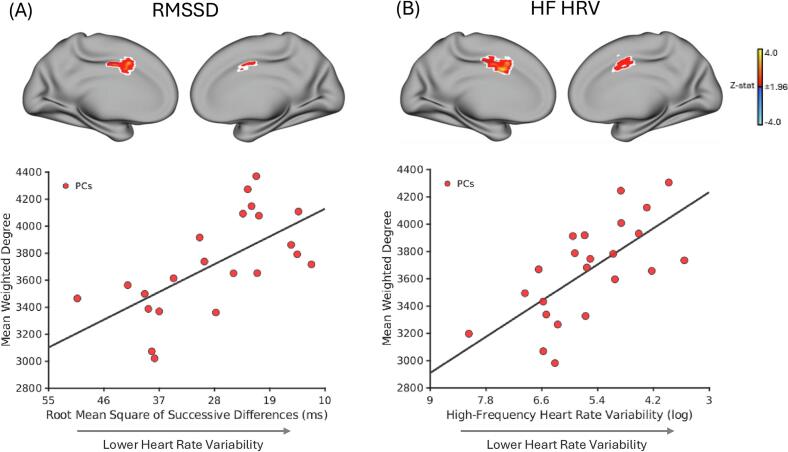
Weighted-degree resting-state functional connectivity (rsFC) correlated with time- and frequency-domain HRV metrics within female psychiatric controls (n = 22 for RMSSD and n = 23 for HF HRV). A) Adjusting for age, head motion, SSRI/SNRI use and β-blocker use, lower root mean square of successive difference (RMSSD) values [a proxy of greater cardiac arousal] were associated with higher weighted-degree rsFC in the bilateral anterior/mid-cingulate cortices (*top* panel). In the *bottom* panel, the scatterplot displays mean weighted-degree values extracted from voxels within the white outlined regions versus RMSSD (ms). B) Adjusting for age, head motion, SSRI/SNRI use and β-blocker use, lower high-frequency heart rate variability (HF HRV) [a proxy of greater cardiac arousal] was associated with higher weighted-degree rsFC in the bilateral anterior/mid-cingulate cortices and the supplementary motor area (*top* panel). In the *bottom* panel, the scatterplot displays mean weighted-degree values extracted from voxels within the white outlined regions versus HF HRV (ln-ms^2^). For *top* panels (A and B), the white outline marks the subset of voxels that remain significant across *post-hoc* corrections additionally adjusting for α-adrenergic blocker use (yes/no) and stimulant medication use (yes/no). Maps show clusters defined by a voxel-wise threshold of *z* > 1.96, corrected for multiple comparisons at the cluster level (*p* < 0.05). Volumetric brain maps are visualized on the cortical surface for display purposes only. The x-axis is reversed to indicate that lower RMSSD and HF HRV correspond to lower HRV as indicated by the arrow [proxy for greater cardiac arousal]. Z-stat, Z-statistic; RMSSD, root mean square of successive differences; SDNN, standard deviation of normal-to-normal intervals.

Within PCs, cardiac autonomic metric–rsFC associations were more spatially restricted. Lower RMSSD was associated with higher weighted-degree rsFC in only the bilateral anterior/mid-cingulate cortices, and these effects remained significant after adjusting for α-adrenergic blocker and stimulant medication use (Fig. 3A). Similarly, lower HF HRV was associated with higher weighted-degree rsFC within the bilateral anterior/mid-cingulate cortices and SMA, with associations remaining significant after both medication adjustments (Fig. 3B). No significant relationships were observed for IBI or SDNN. See **Supplementary** Fig. 3 and Supplementary Table 3 for all significant cardiac autonomic metric-rsFC relationships in female PCs.

## 12 Exploratory post-hoc between-group weighted-degree comparisons within cardiac autonomic-related regions

**Fig. 4:**
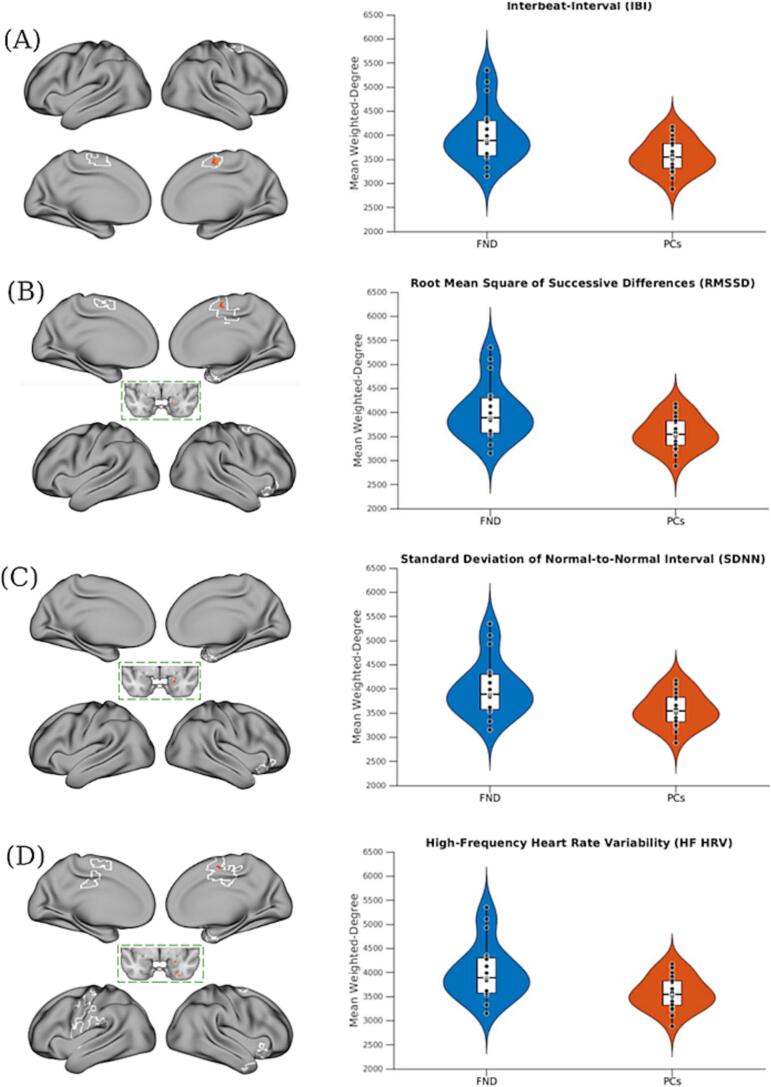
**Between-group *post-hoc* comparison of mean weighted-degree resting-state functional connectivity (rsFC) within IBI, and time- and frequency-domain heart rate variability (HRV)-metrics correlated masks**. **A-D**) Maps on the left show voxels where weighted-degree rsFC differed between patients with functional neurological disorder (FND) and psychiatric controls (PCs), restricted to the subset of voxels that remained significant across all *post-hoc* corrections in the IBI and HRV analyses (Fig. 1 and Fig. 2). Violin plots on the right column show the distribution of mean weighted-degree values extracted from each IBI/HRV metric-correlated masks that remained significant after all *post-hoc* adjustments (white outline in Figs. 1 and 2). FND participants exhibited higher weighted-degree rsFC values within some of these regions compared to PCs (i.e., right supplementary motor area and amygdala)**.** For all display items, individual dots represent participants; white boxes indicate interquartile ranges and medians. Maps show clusters defined by a voxel-wise threshold of z-statistic > 1.96, corrected for multiple comparisons at the cluster level (*p* < 0.05). The volumetric brain map is shown on the cortical surface for display purposes only. RMSSD, root mean square of successive differences; SDNN, standard deviation of normal-to-normal intervals; HF HRV, high frequency heart rate variability.

When investigating potential group-level [FND vs. PC] differences in the brain areas that showed significant correlations between cardiac autonomic metrics and weighted-degree rsFC in patients with FND [as detailed in the preceding results section], *post-hoc* comparisons vs. the PC cohort revealed subtle differences, characterized primarily by modestly higher and more variable rsFC values in the FND cohort. Specifically, patients with FND showed higher weighted-degree rsFC relative to PCs within the right SMA across IBI-, RMSSD-, and HF HRV-related masks, and within the right amygdala across RMSSD-, SDNN-, and HF HRV-related masks (Fig. 4).

## 13 Discussion

In this pilot study, resting cardiac autonomic metrics did not distinguish female patients with FND from age-matched female PCs. Cardiac autonomic metrics also were not significantly associated with self-reported FND symptom severity, somatic symptom burden, affective symptoms, or the magnitude of adverse childhood experiences. However, shorter IBIs (reflecting higher HR) in patients with FND correlated with higher weighted-degree rsFC in the bilateral SMA and primary motor regions. Lower HRV (lower RMSSD, SDNN, HF HRV) was also linked to higher weighted-degree rsFC, primarily within bilateral SMA, cingulate gyrus, temporal pole, and right orbitofrontal cortex, insula, and amygdala. Notably, key associations — particularly within the SMA and right amygdala — remained significant after *post-hoc* adjustments for α-adrenergic blocker and stimulant use, whereas other findings were attenuated. These observations highlight the importance of carefully considering medication use status in multimodal fMRI–autonomic studies, as pharmacologic factors may influence observed brain–autonomic relationships. Exploratory *post-hoc* analyses further clarified that individuals with FND exhibited slightly higher and more variable weighted-degree values in the right SMA and right amygdala compared to PCs – indicating that these areas had a greater [albeit modestly different] contribution to overall functional brain architecture at rest in the FND cohort than in PCs. It should also be noted that in most areas showing relationships between rsFC and cardiac autonomic metrics at rest in patients with FND, the weighted-degree values in those brain regions were comparable to those in PCs. Collectively, these findings suggest that individual-subject cardiac autonomic profiles in FND correlate with the brain organization of multiple rsFC networks previously implicated in the pathophysiology of FND (Drane et al., 2020, Yeo et al., 2011).

In the FND-related autonomic literature, several studies reported elevated resting HR and lower HRV (both often associated with greater cardiac arousal) in patients with FND compared to HCs (Kozlowska et al., 2015, Maurer et al., 2016), whereas others have found no group-level differences in these same metrics (Demartini et al., 2016, Ponnusamy et al., 2012). However, many of these prior studies did not fully account for comorbid depression, anxiety, trauma history, and psychotropic medication use, each of which can impact cardiac autonomic metrics (Alvares et al., 2016, Chalmers et al., 2014, Kemp et al., 2010). In the current study, our controls were well-matched PCs, and we preliminarily show that there may be no distinguishing resting cardiac autonomic profile in adult females with FND at a group-level when compared to a psychiatric (rather than healthy) control group. The absence of between-group differences here does not imply an absence of autonomic relevance. Rather, our findings suggest that select autonomic features are common in individuals with certain psychiatric conditions and FND at the group-level, and also highlight substantial biological heterogeneity within FND – emphasizing the need to take individual differences into account (Diez et al., 2019, Perez et al., 2017). In this context, we also note that our modest sample size may have been underpowered to detect small-to-moderate between-group differences in resting autonomic measures. Larger samples will be needed to clarify whether resting cardiac autonomic differences emerge across groups. Notably, similar heterogeneous observations have been found when investigating potential performance differences on a heartbeat tracking task (assessing the tendency to report the timing of one’s heartbeats with accuracy), with some FND cohort studies showing between-group differences in relation to HCs (Koreki et al., 2020, Ricciardi et al., 2014) and others showing similar performance across groups (Jungilligens et al., 2020, Pick et al., 2020, Sojka et al., 2021). Relatedly, differences in heartbeat tracking accuracy across patients with FND correlated with individual differences in cingulo-insular activation (Sojka et al., 2025). These results underscore that various physiological measurements in FND may not be best conceptualized as standalone biomarkers, but rather by *how* physiological measurements (e.g., cardiac autonomic metrics at rest) relate to other biological systems—such as functional brain network architecture.

Across the IBI and HRV within-group analyses in patients with FND, the SMA emerged as a consistently implicated region, with both shorter IBI [higher HR] and lower HRV associated with higher rsFC weighted-degree values. Both shorter IBIs and lower HRV may reflect greater cardiac arousal. These observations are noteworthy given that several fMRI studies have identified a role for the SMA and its connections in the pathophysiology of FND. For example, task-based fMRI studies have shown increased SMA activation during recall of affectively charged autobiographical events (Aybek et al., 2014), increased SMA recruitment during exposure to normatively negative emotional stimuli (Aybek et al., 2015), and abnormal SMA activity during motor preparation even when no movement was performed (Voon et al., 2011). In a pilot real-time fMRI study, greater subjective agency in patients with FND were also associated with upregulation of SMA and right temporoparietal junction activity (Müller et al., 2025). Prior work from our group has also shown that rsFC relationships between the anterior insula and the SMA relate to individual differences in functional neurological symptom severity (Diez et al., 2019). SMA rsFC profiles, as measured using BOLD signal variability, have also shown prognostic relevance in patients with FND (Schneider et al., 2024). Neurobiologically, the SMA is a premotor hub theorized to be involved in internally-generated action planning and motor initiation, and it is functionally connected with the somatomotor and salience networks (Hallett, 2022). As such, Voon et al (2011) proposed that during higher physiological arousal there may be a disruption in the action-selection process that is in part mediated through the SMA (Voon et al., 2011). Convergent pediatric studies have similarly implicated somatomotor regions – particularly the SMA – with reports of increased activity and grey matter alterations in children and adolescents with FND (Kozlowska et al., 2017b, Kozlowska et al., 2018). In relation to the findings of this study, it is important to highlight that the SMA is a cortical component of the CAN (Beissner et al., 2013, Valenza et al., 2019). Given the results of this present study, we speculate that the SMA may be one hub through which cardiac autonomic signals influence motor expression patterns in patients with FND.

Beyond the SMA, individual differences in weighted-degree relationships to HRV metrics were also observed in the right amygdala, anterior insula and lateral orbitofrontal cortex. Specifically, lower HRV correlated with higher centrality of these brain areas in the overall functional architecture; conversely, those patients with FND *and* relatively higher HRV indices showed lower amygdala, anterior insula and orbitofrontal cortex influence on their overall functional brain architecture. These brain regions overlap in their contributions to the CAN, the allostatic-interoceptive network, and several canonical resting-state functional networks (i.e., salience and limbic networks) (Kleckner et al., 2017, Quadt et al., 2022, Yeo et al., 2011). Given that motor control areas [including but not limited to the SMA] have shown increased functional connectivity to the amygdala and insula across task and rs-fMRI studies in patients with FND (Aybek et al., 2015, Aybek et al., 2014, Diez et al., 2019, Li et al., 2015b, Voon et al., 2011), the findings of this present work suggest that individual differences in cardiac autonomic metrics could relate to the role of the amygdala and anterior insula in overall functional brain organization. Interestingly, prior research has shown that moment-to-moment fluctuations in HRV may covary with dynamic resting-state connectivity of salience network hubs, including the amygdala and dorsal anterior cingulate cortex – suggesting that in addition to the static rsFC approach used here, dynamic rsFC analyses should also be pursued in larger-scale efforts (Chang et al., 2013, Schneider et al., 2024). Additionally, while we did not identify correlations between cardiac autonomic metrics at rest and childhood maltreatment burden [potentially due to modest sample size], such early-life factors can influence the autonomic nervous system over the long-term – including promoting tendencies towards having lower HRV (Stevens et al., 2023, Stürmer et al., 2025). This literature is noteworthy given “dose-dependent relationships” previously reported between the magnitude of childhood physical abuse burden and the rsFC strength relationships between the amygdala and insula to the primary motor cortex (Diez et al., 2021). Taken together, these findings are consistent with the notion that in some patients with FND, hubs within the CAN, the allostatic-interoceptive network, and the salience network show altered contributions to the overall functional network architecture.

The inclusion of a psychiatric comparison group also provides important insight into the biological heterogeneity of FND. While associations between weighted-degree and cardiac autonomic metrics were present in both groups, they were more spatially extensive in FND, whereas PCs showed a more limited pattern centered mainly on the anterior/mid-cingulate cortices. Exploratory *post-hoc* analyses further support this interpretation: within autonomic-associated regions, patients with FND exhibited slightly higher and more variable weighted-degree values in the right SMA and right amygdala compared to PCs. These between-group comparisons were conducted within regions identified from the FND cohort and should therefore be interpreted as exploratory pending independent replication. On inspection of the data, brain-autonomic relationships in patients with FND appear more robust and widespread in part because of the larger between-subject heterogeneity observed in this population.

This pilot study has several noteworthy limitations. First, the sample size is modest, and as such, the results should be considered preliminary. Although a whole-brain cluster-wise correction was applied within each voxel-wise model, we did not adjust for multiple tests across the broader set of analyses performed, and findings should therefore be interpreted with caution. Additionally, the absence of a healthy control group limits interpretation of between-group findings, as it is unclear whether FND and PC cohorts in this study exhibit similar or distinct autonomic profiles relative to healthy individuals (Wang et al., 2025). The focus on only female participants limits the generalizability of our findings to males with FND, underscoring the need to explore sex differences in brain-autonomic relationships in patients with FND, as well as the need to investigate potential differences across FND phenotypes. Furthermore, cardiac autonomic metrics were assessed at rest; while this approach eliminates task-related confounds, it also does not capture dynamic autonomic responsivity to a provocation. We also did not systematically control for contextual physiological factors known to influence HRV − such as hormonal status and amount/timing of caffeine intake − which may contribute to variability in autonomic estimates. Similarly, respiration was not directly measured and therefore could not be modeled as a control variable for the frequency-domain HRV metric (i.e., HF HRV). Future studies incorporating respiratory monitoring and more comprehensive standardization of factors influencing cardiac autonomic metrics would allow for more specific interpretation of HRV findings. Future research could also benefit from simultaneous in-scanner cardiac autonomic metrics acquisition, allowing for concurrent characterization of both resting-state brain connectivity and cardiac autonomic relationships. Additional multimodal, longitudinal, and provocation-based studies are needed to comprehensively delineate the role of the autonomic nervous system in the pathophysiology of FND; as such, we defer more definitive inferences regarding specific sympathetic and parasympathetic impacts on cardiac arousal metrics to future research. It is important to note that measures of IBI and HRV do not always cohere, meaning that individuals may not consistently have both slower IBI (greater resting HR) and lower resting HRV, or vice versa, in part because these metrics are influenced by distinct autonomic inputs. Specifically, HR is influenced by both sympathetic and parasympathetic effects, and not always in opposite directions (Berntson et al., 1991, Berntson et al., 1993, Berntson et al., 1994), whereas HRV is predominantly a function of parasympathetic effects, although here too, other influences, such as respiratory rate and depth, can confound simple inferences (Quigley et al., 2024). Further, different metrics of peripheral physiological arousal also do not always relate in a simple one-to-one way with metrics of central nervous system arousal (Satpute et al., 2019). Finally, the current cross-sectional study design cannot adjudicate whether cardiac autonomic–brain network associations represent a predisposing vulnerability, a perpetuating factor, and/or compensatory mechanisms (among other possibilities). Large-scale multimodal brain imaging efforts, along with a more comprehensive sampling of autonomic parameters (e.g., characterization of autonomic variables beyond cardiac metrics), will be important to replicate and extend the findings of this initial study (Roberts et al., 2012, Roberts et al., 2020).

In summary, this pilot study provides early-phase evidence that individual differences in cardiac autonomic metrics relate to large-scale brain network architecture in FND. Moving forward, multimodal physiological sampling — including the dual acquisition of brain imaging and peripheral autonomic measures — may provide a more complete and nuanced understanding of the pathophysiology of FND.

## CRediT authorship contribution statement

**Cristina Bleier:** Writing – original draft, Visualization, Methodology, Formal analysis, Data curation, Conceptualization. **Andrew J. Guthrie:** Writing – review & editing, Visualization, Methodology, Formal analysis, Data curation, Conceptualization. **Jessica Ranford:** Writing – review & editing, Data curation, Conceptualization. **Julie MacLean:** Writing – review & editing, Methodology, Data curation. **Ellen Godena:** Writing – review & editing, Methodology, Data curation. **Julie Maggio:** Writing – review & editing, Methodology, Data curation. **Sara A. Finkelstein:** Writing – review & editing, Formal analysis, Data curation, Conceptualization. **Ibai Diez:** Writing – review & editing, Methodology, Formal analysis. **Christiana Westlin:** Writing – review & editing, Supervision, Methodology, Formal analysis. **Karen S. Quigley:** Writing – review & editing, Supervision, Methodology, Data curation, Conceptualization. **David L. Perez:** Writing – review & editing, Writing – original draft, Supervision, Methodology, Funding acquisition, Formal analysis, Data curation, Conceptualization.

## 14 Funding

This project was supported by the National Institute of Mental Health R01MH125802.

## Declaration of competing interest

K.S.Q. is a consulting editor for *Psychophysiology*, *Affective Science*, and *Biological Psychology*. D.L.P. has received honoraria for continuing medical education lectures in FND, royalties from Springer for a functional movement disorder textbook and honoraria from Elsevier for a functional neurological disorder textbook; is on the editorial boards of *The Journal of Neuropsychiatry and Clinical Neurosciences* (paid), *NeuroImage Clinical* (paid), *Brain and Behavior*, *Epilepsy & Behavior*, *Cognitive and Behavioral Neurology,* and *General Hospital Psychiatry*; has received funding from the Sidney R. Baer Jr. Foundation and the Warren Alpert Foundation unrelated to this work; and is on the FND Society Board and American Neuropsychiatric Association Advisory Council. All other authors report no known financial interests or personal relationships that could have appeared to influence the work reported in this paper.

## Data Availability

For qualified researchers, analysis code and de-identified data pertaining only to study results can be made available upon reasonable request and following approval by the local internal review board.
