# Supplementary material for: Relationships between brain functional connectivity and resting cardiac autonomic profiles in functional neurological disorder: A pilot study
Source: Neuroimage Clin. 2026 Apr 22;50:103996. doi: 10.1016/j.nicl.2026.103996 (PMC13157166; doi:10.1016/j.nicl.2026.103996)
Supplement: Supplementary Data 3 [file mmc3.docx]

**Supplementary Table 3. A complete description of statistically significant findings across all of the resting-state weighted-degree – cardiac autonomic analyses.**

*IBI (FND)*

| Cluster | Size(mm3) | X | Y | Z | | Zstat_peak | Zstat_mean | Region |
| --- | --- | --- | --- | --- | --- | --- | --- | --- |
| 1 | 6831 | 32.5 | -14.5 | 60.5 | | 3.62 | 2.39 | ctx-rh-superiorfrontal |
|  | | | | |  | | | ctx-rh-precentral |
|  |  |  |  |  |  |  |  | ctx-lh-superiorfrontal |
|  |  |  |  |  |  |  |  | ctx-rh-caudalmiddlefrontal |
|  |  |  |  |  |  |  |  |  |

*RMSSD (FND)*

| Cluster | | Size(mm3) | X | Y | Z | Zstat_peak | Zstat_mean | Region | |
| --- | --- | --- | --- | --- | --- | --- | --- | --- | --- |
| 1 | 6777 | | 8.5 | 0.5 | 57.5 | 3.97 | 2.39 | ctx-rh-superiorfrontal | |
|  | | | | | | | | | ctx-rh-posteriorcingulate |
|  |  |  |  |  |  |  |  |  | ctx-lh-superiorfrontal |
|  |  |  |  |  |  |  |  |  | ctx-lh-posteriorcingulate |
|  |  |  |  |  |  |  |  |  | ctx-rh-paracentral |
|  |  |  |  |  |  |  |  |  |  |
| 2 | 7668 | | 23.5 | 3.5 | -38.5 | 3.34 | 2.34 | ctx-rh-lateralorbitofrontal | |
|  | | | | | | | | | ctx-rh-superiortemporal |
|  |  |  |  |  |  |  |  |  | ctx-rh-insula |
|  |  |  |  |  |  |  |  |  | ctx-rh-temporalpole |
|  |  |  |  |  |  |  |  |  | rh-amygdala |
|  |  |  |  |  |  |  |  |  | ctx-rh-parsorbitalis |
|  |  |  |  |  |  |  |  |  | ctx-rh-fusiform |
|  |  |  |  |  |  |  |  |  |  |
| 3 | 4833 | | -57.5 | -5.5 | 15.5 | 2.91 | 2.22 | ctx-lh-precentral | |
|  | | | | | | | | | ctx-lh-postcentral |
|  |  |  |  |  |  |  |  |  | ctx-lh-superiortemporal |
|  |  |  |  |  |  |  |  |  |  |

*SDNN (FND)*

| Cluster | Size(mm3) | X | Y | Z | Zstat_peak | Zstat_mean | Region |
| --- | --- | --- | --- | --- | --- | --- | --- |
| 1 | 7317 | 8.5 | 0.5 | 57.5 | 4.02 | 2.46 | ctx-rh-superiorfrontal |
|  | | | | | | | ctx-rh-posteriorcingulate |
|  |  |  |  |  |  |  | ctx-lh-superiorfrontal |
|  |  |  |  |  |  |  | ctx-lh-posteriorcingulate |
|  |  |  |  |  |  |  | ctx-rh-paracentral |
|  |  |  |  |  |  |  |  |
| 2 | 4995 | 23.5 | -44.5 | -47.5 | 4.01 | 2.47 | ctx-rh-cerebellum |
|  | | | | | | | ctx-lh cerebellum |
|  |  |  |  |  |  |  | brain-stem |
|  |  |  |  |  |  |  |  |
| 3 | 9963 | -33.5 | 3.5 | 48.5 | 3.98 | 2.36 | ctx-lh-precentral |
|  | | | | | | | ctx-lh-caudalmiddlefrontal |
|  |  |  |  |  |  |  | ctx-lh-postcentral |
|  |  |  |  |  |  |  | ctx-lh-superiortemporal |
|  |  |  |  |  |  |  | ctx-lh-supramarginal |
|  |  |  |  |  |  |  | ctx-lh-superiorfrontal |
|  |  |  |  |  |  |  |  |
| 4 | 10422 | 44.5 | 15.5 | -41.5 | 3.81 | 2.4 | ctx-rh-lateralorbitofrontal |
|  | | | | | | | ctx-rh-superiortemporal |
|  |  |  |  |  |  |  | ctx-rh-middletemporal |
|  |  |  |  |  |  |  | ctx-rh-temporalpole |
|  |  |  |  |  |  |  | ctx-rh-parsorbitalis |
|  |  |  |  |  |  |  | ctx-rh-insula |
|  |  |  |  |  |  |  | rh-amygdala |
|  |  |  |  |  |  |  | ctx-rh-parstriangularis |
|  |  |  |  |  |  |  |  |
| 5 | 8856 | 53.5 | -32.5 | -5.5 | 3.62 | 2.34 | ctx-rh-middletemporal |
|  | | | | | | | ctx-rh-superiortemporal |
|  |  |  |  |  |  |  | ctx-rh-postcentral |
|  |  |  |  |  |  |  | ctx-rh-insula |
|  |  |  |  |  |  |  | ctx-rh-precentral |
|  |  |  |  |  |  |  | ctx-rh-bankssts |
|  |  |  |  |  |  |  | rh-thalamus |
|  |  |  |  |  |  |  | ctx-rh-transversetemporal |
|  |  |  |  |  |  |  |  |
| 6 | 20547 | 8.5 | 39.5 | 30.5 | 3.52 | 2.33 | ctx-rh-superiorfrontal |
|  | | | | | | | ctx-rh-rostralmiddlefrontal |
|  |  |  |  |  |  |  | ctx-lh-superiorfrontal |
|  |  |  |  |  |  |  | ctx-lh-rostralmiddlefrontal |
|  |  |  |  |  |  |  | ctx-rh-rostralanteriorcingulate |
|  |  |  |  |  |  |  | ctx-lh-rostralanteriorcingulate |
|  |  |  |  |  |  |  | ctx-rh-caudalanteriorcingulate |
|  |  |  |  |  |  |  |  |

*HF-HRV (FND)*

| Cluster | Size(mm3) | X | Y | Z | Zstat_peak | Zstat_mean | Region |
| --- | --- | --- | --- | --- | --- | --- | --- |
| 1 | 4941 | -39.5 | -62.5 | -11.5 | 4.19 | 2.49 | ctx-lh-fusiform |
|  | | | | | | | ctx-lh-cerebellum |
|  |  |  |  |  |  |  | ctx-lh-lateraloccipital |
|  |  |  |  |  |  |  | ctx-lh-lingual |
|  |  |  |  |  |  |  |  |
| 2 | 15282 | 50.5 | 15.5 | -35.5 | 4.1 | 2.44 | ctx-rh-lateralorbitofrontal |
|  | | | | | | | ctx-rh-superiortemporal |
|  |  |  |  |  |  |  | ctx-rh-insula |
|  |  |  |  |  |  |  | ctx-rh-middletemporal |
|  |  |  |  |  |  |  | ctx-rh-parsorbitalis |
|  |  |  |  |  |  |  | ctx-rh-temporalpole |
|  |  |  |  |  |  |  | rh-amygdala |
|  |  |  |  |  |  |  | ctx-rh-parsopercularis |
|  |  |  |  |  |  |  | ctx-rh-inferiortemporal |
|  |  |  |  |  |  |  | ctx-rh-parstriangularis |
|  |  |  |  |  |  |  | rh-putamen |
|  |  |  |  |  |  |  | ctx-rh-fusiform |
|  |  |  |  |  |  |  |  |
| 3 | 9639 | 11.5 | -11.5 | 42.5 | 3.9 | 2.52 | ctx-rh-posteriorcingulate |
|  | | | | | | | ctx-rh-superiorfrontal |
|  |  |  |  |  |  |  | ctx-lh-posteriorcingulate |
|  |  |  |  |  |  |  | ctx-lh-superiorfrontal |
|  |  |  |  |  |  |  | ctx-rh-paracentral |
|  |  |  |  |  |  |  | ctx-lh-paracentral |
|  |  |  |  |  |  |  |  |
| 4 | 13878 | 17.5 | 63.5 | -2.5 | 3.84 | 2.37 | ctx-rh-rostralmiddlefrontal |
|  | | | | | | | ctx-rh-superiorfrontal |
|  |  |  |  |  |  |  | ctx-lh-superiorfrontal |
|  |  |  |  |  |  |  | ctx-rh-rostralanteriorcingulate |
|  |  |  |  |  |  |  | ctx-lh-rostralanteriorcingulate |
|  |  |  |  |  |  |  | ctx-rh-caudalanteriorcingulate |
|  |  |  |  |  |  |  |  |
| 5 | 13392 | -48.5 | 15.5 | 3.5 | 3.73 | 2.41 | ctx-lh-precentral |
|  | | | | | | | ctx-lh-postcentral |
|  |  |  |  |  |  |  | ctx-lh-parsopercularis |
|  |  |  |  |  |  |  | ctx-lh-supramarginal |
|  |  |  |  |  |  |  | ctx-lh-superiortemporal |
|  |  |  |  |  |  |  |  |
| 6 | 7452 | 53.5 | -32.5 | -5.5 | 3.53 | 2.38 | ctx-rh-middletemporal |
|  | | | | | | | ctx-rh-superiortemporal |
|  |  |  |  |  |  |  | ctx-rh-postcentral |
|  |  |  |  |  |  |  | ctx-rh-precentral |
|  |  |  |  |  |  |  | ctx-rh-transversetemporal |
|  |  |  |  |  |  |  |  |

*RMSSD (PC)*

| Cluster | Size(mm3) | X | Y | Z | Zstat_peak | Zstat_mean | Region |
| --- | --- | --- | --- | --- | --- | --- | --- |
| 1 | 1350 | -0.5 | -2.5 | 39.5 | 4.08 | 2.95 | ctx-lh-posteriorcingulate |
|  | | | | | | | ctx-rh-posteriorcingulate |
|  |  |  |  |  |  |  |  |

*HF-HRV (PC)*

| Cluster | Size(mm3) | X | Y | Z | Zstat_peak | Zstat_mean | Region |
| --- | --- | --- | --- | --- | --- | --- | --- |
| 1 | 2079 | -0.5 | -2.5 | 39.5 | 3.81 | 2.8 | ctx-lh-posteriorcingulate |
|  | | | | | | | ctx-rh-posteriorcingulate |
|  |  |  |  |  |  |  | ctx-lh-caudalanteriorcingulate |
|  |  |  |  |  |  |  |  |
