# Supplementary figures and images for: Relationships between brain functional connectivity and resting cardiac autonomic profiles in functional neurological disorder: A pilot study
Source: Neuroimage Clin. 2026 Apr 22;50:103996. doi: 10.1016/j.nicl.2026.103996 (PMC13157166; doi:10.1016/j.nicl.2026.103996)

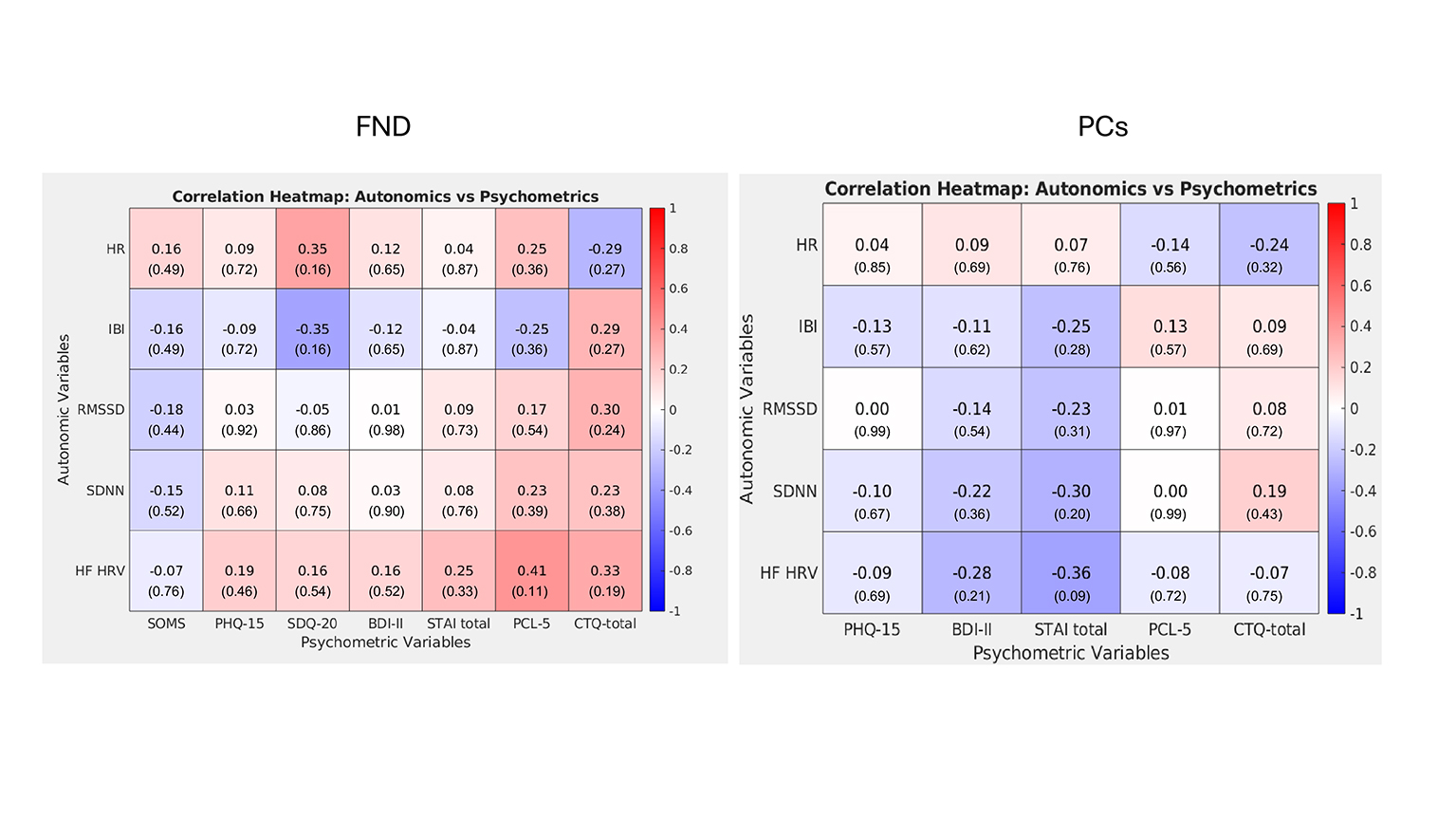

Supplement: Supplementary Figure 4 [file mmc4.jpg]

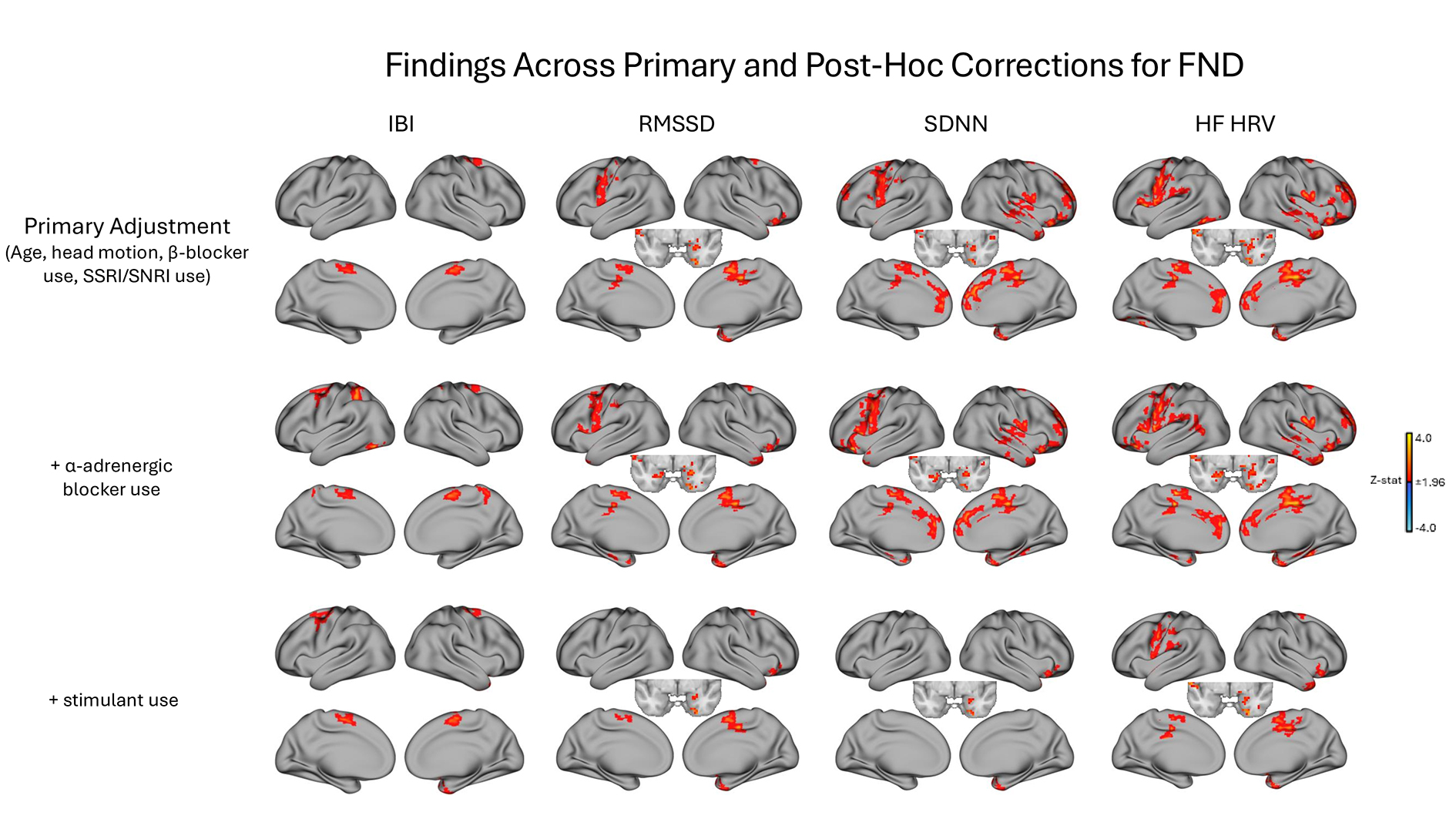

Supplement: Supplementary Figure 5 [file mmc5.jpg]

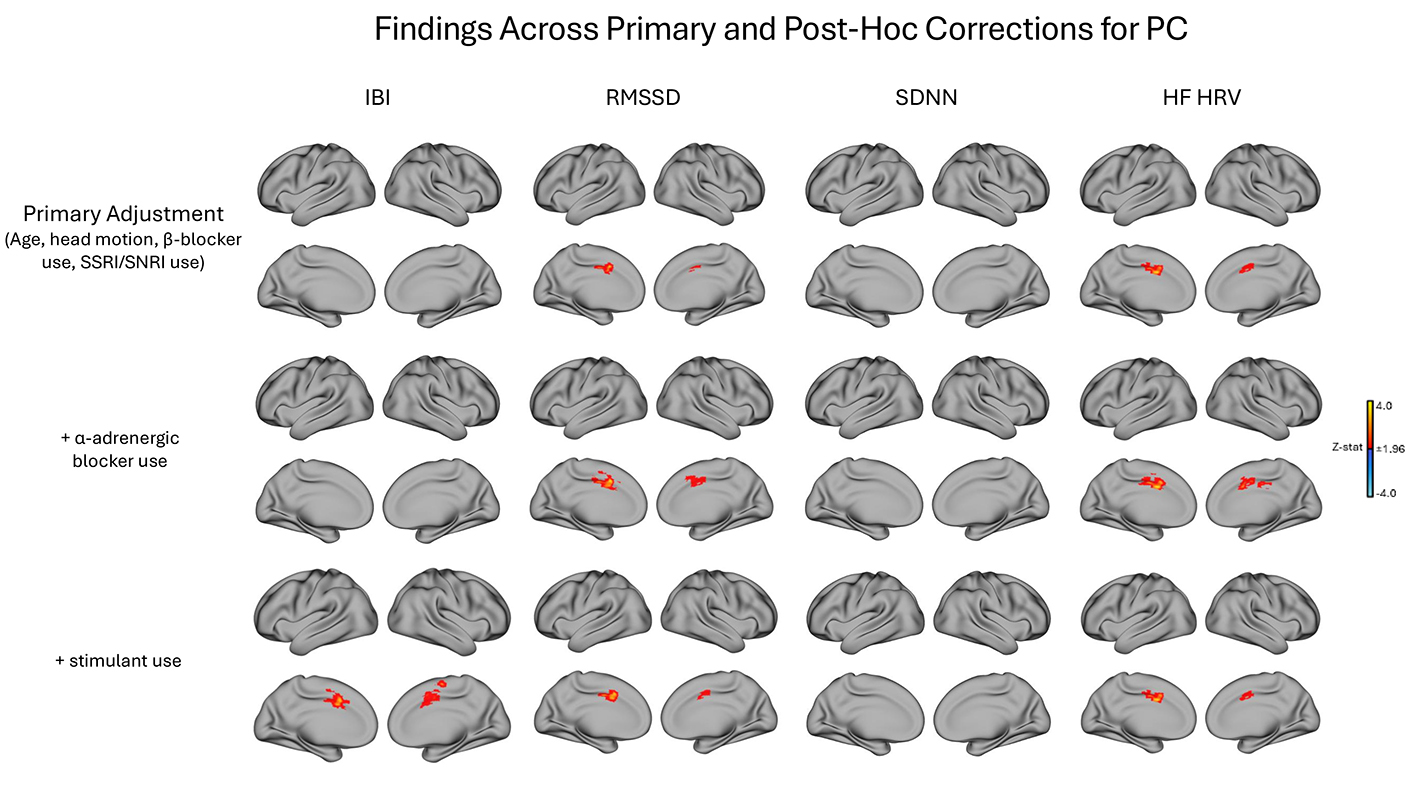

Supplement: Supplementary Figure 6 [file mmc6.jpg]
